# Identification of biomarkers associated with the feed efficiency by metabolomics profiling: results from the broiler lines divergent for high or low abdominal fat content

**DOI:** 10.1186/s40104-022-00775-3

**Published:** 2022-11-10

**Authors:** Zhiyong Su, Xue Bai, Haoyu Wang, Shouzhi Wang, Chong Chen, Fan Xiao, Huaishun Guo, Haihe Gao, Li Leng, Hui Li

**Affiliations:** 1 Key Laboratory of Chicken Genetics and Breeding, Ministry of Agriculture and Rural Affairs, Harbin, 150030 China; 2 Key Laboratory of Animal Genetics, Breeding and Reproduction, Education Department of Heilongjiang Province, Harbin, 150030 China; 3 College of Animal Science and Technology, Northeast Agricultural University, Harbin, 150030 China; 4 Fujian Sunnzer Biotechnology Development Co., Ltd, Guangze, 354100 China

**Keywords:** Biomarker, Broiler, Feed efficiency, Metabolomics

## Abstract

**Background:**

Improving feed efficiency (FE) is one of the main objectives in broiler breeding. It is difficult to directly measure FE traits, and breeders hence have been trying to identify biomarkers for the indirect selection and improvement of FE traits. Metabolome is the "bridge" between genome and phenome. The metabolites may potentially account for more of the phenotypic variation and can suitably serve as biomarkers for selecting FE traits. This study aimed to identify plasma metabolite markers for selecting high-FE broilers. A total of 441 birds from Northeast Agricultural University broiler lines divergently selected for abdominal fat content were used to analyze plasma metabolome and estimate the genetic parameters of differentially expressed metabolites.

**Results:**

The results identified 124 differentially expressed plasma metabolites (*P* < 0.05) between the lean line (high-FE birds) and the fat line (low-FE birds). Among these differentially expressed plasma metabolites, 44 were found to have higher positive or negative genetic correlations with FE traits (|*r*_g_| ≥ 0.30). Of these 44 metabolites, 14 were found to display moderate to high heritability estimates (*h*^2^ ≥ 0.20). However, among the 14 metabolites, 4 metabolites whose physiological functions have not been reported were excluded. Ultimately, 10 metabolites were suggested to serve as the potential biomarkers for breeding the high-FE broilers. Based on the physiological functions of these metabolites, reducing inflammatory and improving immunity were proposed to improve FE and increase production efficiency.

**Conclusions:**

According to the pipeline for the selection of the metabolite markers established in this study, it was suggested that 10 metabolites including 7-ketocholesterol, dimethyl sulfone, epsilon-(gamma-glutamyl)-lysine, gamma-glutamyltyrosine, 2-oxoadipic acid, *L*-homoarginine, testosterone, adenosine 5'-monophosphate, adrenic acid, and calcitriol could be used as the potential biomarkers for breeding the "food-saving broilers".

## Introduction

Livestock farming serves to provide high-quality proteins to humans but requires the consumption of a lot of grain resources, intensifying the contradiction of "Competition for Food between Human and Animal" [1]. Feeding livestock with high feed efficiency (FE) can beneficially reduce food consumption, saving the expenditure on livestock production and contributing to the sustained development of the environment [2, 3]. Breeders have always focused on improving FE of broilers [4]. The direct selection of FE traits has been the most effective method for improving FE. However, direct selection is based on accurately measuring feed intake, and implementing this method in breeding farms is difficult [5]. Breeders are attempting to find easily measurable indicators for the rapid and affordable estimation of the individual FE traits.

The metabolome is often regarded as a bridge between the genome and the phenome [6]. As downstream of the gene regulatory network and the protein interaction network, metabolites may provide detailed biological terminal information on the life process [7]. In recent years, metabolomics has evolved as an increasingly popular "omics" approach for revealing the relationship between genetics and phenotypes [7]. Researchers can discover novel biomarkers by analyzing the changes in metabolite expression profiling to further understand the vital metabolic pathways associated with the traits. Emerging evidence indicated the use of metabolites as metabolic markers for selecting FE traits in mammals. A study by Novais et al. evaluated the feasibility of predicting FE traits through serum metabolomics in young Nellore cattle [8]. This study found the retinal, progesterone, and stearic acid as suitable biomarkers for indirectly selecting FE traits, and the vitamin A metabolism pathway was identified as an important pathway related to FE traits [8]. A study by Carmelo et al. found choline and pyridoxamine as the hub-metabolites related to FE traits in Duroc pigs, at the same time, cholesterol sulfate, thiamine, *L*-methionine, and chenodeoxycholate were identified as the hub-metabolites related to FE traits in Landrace pigs, suggesting that these metabolites served as biomarkers for selecting FE traits [9]. Therefore, metabolomics can not only help investigate the relationship between metabolites and phenotypes but also can be used for identifying biomarkers for selecting high-FE animals. However, the metabolomics study of FE traits in broilers remains poorly understood.

The Northeast Agricultural University broiler lines divergently selected for abdominal fat content (NEAUHLF) were selected based on the abdominal fat percentage (AFP) and plasma concentration of very low-density lipoprotein (VLDL) since 1996 [10]. A previous study identified the feed conversion ratio (FCR) and residual feed intake (RFI) to be significantly higher in the fat line than in the lean line [11]. In the present study, firstly, the differences in plasma metabolome between the lean and fat lines were investigated. Subsequently, the genetic parameters of the concentrations of differentially expressed plasma metabolites were estimated. Finally, the metabolite biomarkers that could be potentially used to select high-FE broilers were identified.

## Materials and methods

### Experimental population

This study used 441 birds from the 23^rd^ generation of the NEAUHLF lines, which were selected based on AFP and plasma concentration of VLDL [10]. All the birds were housed in the similar environmental conditions and had free access to feed and water up to 7 weeks from hatching. The commercial corn-soybean-based diets, meeting all the requirements of the National Research Council (NRC, 1994) [12], were used in the study. The birds received a starter feed (3000 kcal ME/kg and 210 g/kg CP) from hatching to 3 weeks. Then, the birds were fed a grower diet (3100 kcal ME/kg and 190 g/kg CP) from 4 weeks to slaughter. Each bird was placed in an individual cage at 27 days of age, and the individual feed intake (FI) was recorded at 29—49 days of age.

### Trait measurement

The total FI of each bird was recorded from 4 weeks old to 7 weeks old. The body weights were measured at 4 weeks (BW4) and 7 weeks (BW7) of age. The gain in body weights (BWG) was calculated by subtracting the BW4 from BW7. The metabolic mid-test body weight (MMBW) was calculated by taking the 0.75^th^ power of the middle body weight during the specific period [(*BW4* + *BW7*)/2]. The FCR and RFI were calculated using the following equations:

$$FCR=FI/BWG$$

$$RFI=FI-\left(b_0+b_1MMBW+b_2BWG\right)$$

where *b*_0_ is the intercept and *b*_1_, *b*_2_ are partial regression coefficients of *FI* for *MMBW* and *BWG*, respectively.

### Metabolites extraction

The whole-blood samples were collected and immediately centrifuged at 3000 × *g* for 10 min at 25 °C. The supernatants (100 μL plasma) were placed in the EP tubes and resuspended in prechilled 80% methanol by vortexing. Then, the samples were incubated on ice for 5 min and centrifuged at 15,000 × *g* for 20 min at 4 °C. The supernatant was diluted to a final concentration of 53% methanol using LC–MS grade water. The samples were transferred to a fresh EP tube and centrifuged at 15,000 × *g* for 20 min at 4 °C. Finally, the supernatant was injected into the LC–MS/MS system for subsequent analysis.

### UHPLC/MS analysis

The UHPLC/MS was analyzed using a Vanquish UHPLC system (ThermoFisher, Germany) coupled with an Orbitrap Q Exactive TMHF-X mass spectrometer (Thermo Fisher, Germany) in Novogene Co., Ltd. (Beijing, China). The samples were injected onto a Hypesil Gold column (100 mm × 2.1 mm, 1.9 μm) using a 17-min linear gradient at a flow rate of 0.2 mL/min. The eluents for the positive polarity mode were eluent A (0.1% formic acid in water) and eluent B (methanol). The eluents for the negative polarity mode were eluent A (5 mmol/L ammonium acetate, pH 9.0) and eluent B (methanol). The solvent gradient was set as follows: 2% B, 1.5 min; 2%—100% B, 12.0 min; 100% B, 14.0 min; 100%—2% B, 14.0 min; and 2% B, 17.0 min. The Q Exactive™ HF-X mass spectrometer was operated in the positive/negative polarity mode using a spray voltage of 3.2 kV, capillary temperature of 320 °C, sheath gas flow rate of 40 arb, and aux gas flow rate of 10 arb.

The raw data files generated by UHPLC/MS were analyzed using the Compound Discoverer 3.1 (CD 3.1, ThermoFisher) for peak alignment, peak picking, and quantitation for each metabolite. The main parameters were set as follows: retention time tolerance, 0.2 min; actual mass tolerance, 5 ppm; signal intensity tolerance, 30%; and signal/noise ratio, 3. Subsequently, the peak intensities were normalized to the total spectral intensity and the normalized data were used for predicting the molecular formula based on the additive ions, molecular ion peaks, and fragment ions. Then, the peaks were matched with the mzCloud, mzVault, MassList, and ChemSpider databases for obtaining accurate qualitative and relative quantitative results [13, 14]. These metabolites were annotated using the human metabolome database (HMDB) [9], ignoring the metabolites that did not correspond to the HMDB.

### Statistical analysis

The differences among the groups were explored using the partial least square-discriminant analysis (PLS-DA). The quality of PLS-DA model was assessed using the goodness of fit (R^2^) and goodness of prediction (Q^2^) in sevenfold cross validation [8]. The variable importance in projection (VIP) was considered for screening out the discriminant metabolites. The statistical significance (*P* value) was calculated using the Student’s *t*-test. The metabolites with VIP > 1 and *P* < 0.05 were considered as differential metabolites between the lean and fat lines [15]. For heatmaps, the data were normalized using the log10 of the concentrations of differential metabolites and plotted using the Pheatmap package in the R (version 4.1.2) environment.

The genetic parameters, including the heritability of the metabolites as well as the genetic and phenotypic correlations between these metabolites and FE trait indices (FCR and RFI), were estimated using the ASReml (version 4.0) software, with line and sex treated as fixed effects. The animal model used for estimating the genetic parameters was as follows:

$$\boldsymbol Y=\boldsymbol X\boldsymbol b+\boldsymbol Z\boldsymbol a+\boldsymbol e$$

where ***Y*** represents the vector of the concentration of the plasma metabolites or the phenotypic value of the FE trait indices; ***b*** represents the vector of the fixed effect including the population mean, sex effects, and line effects; ***a*** represents the vector of the random effects including the genetic effects; ***e*** represents the vector of the random residual effects; and ***X*** and ***Z*** are the incidence matrices for ***b*** and ***a***. The random-effects ***a*** and ***e*** were assumed to follow the normal distributions with a mean of 0. The variances of ***a*** and ***e*** were assumed to be Var(***a***) = ***A****g* and Var(***e***) = ***I****r*, respectively, in which ***A*** represents the numerator relationship matrix of all the animals in the pedigree file, *g* is the additive genetic variance, ***I*** is the identity matrix, and *r* is the residual variance [16]. According to the recommendation by Dong et al., the thresholds in this study for high genetic correlation and moderate to high heritability were set to be greater than 0.30 and 0.20, respectively [17].

## Results

### Metabolite profile differences between the lean and fat lines

**Fig. 1:**
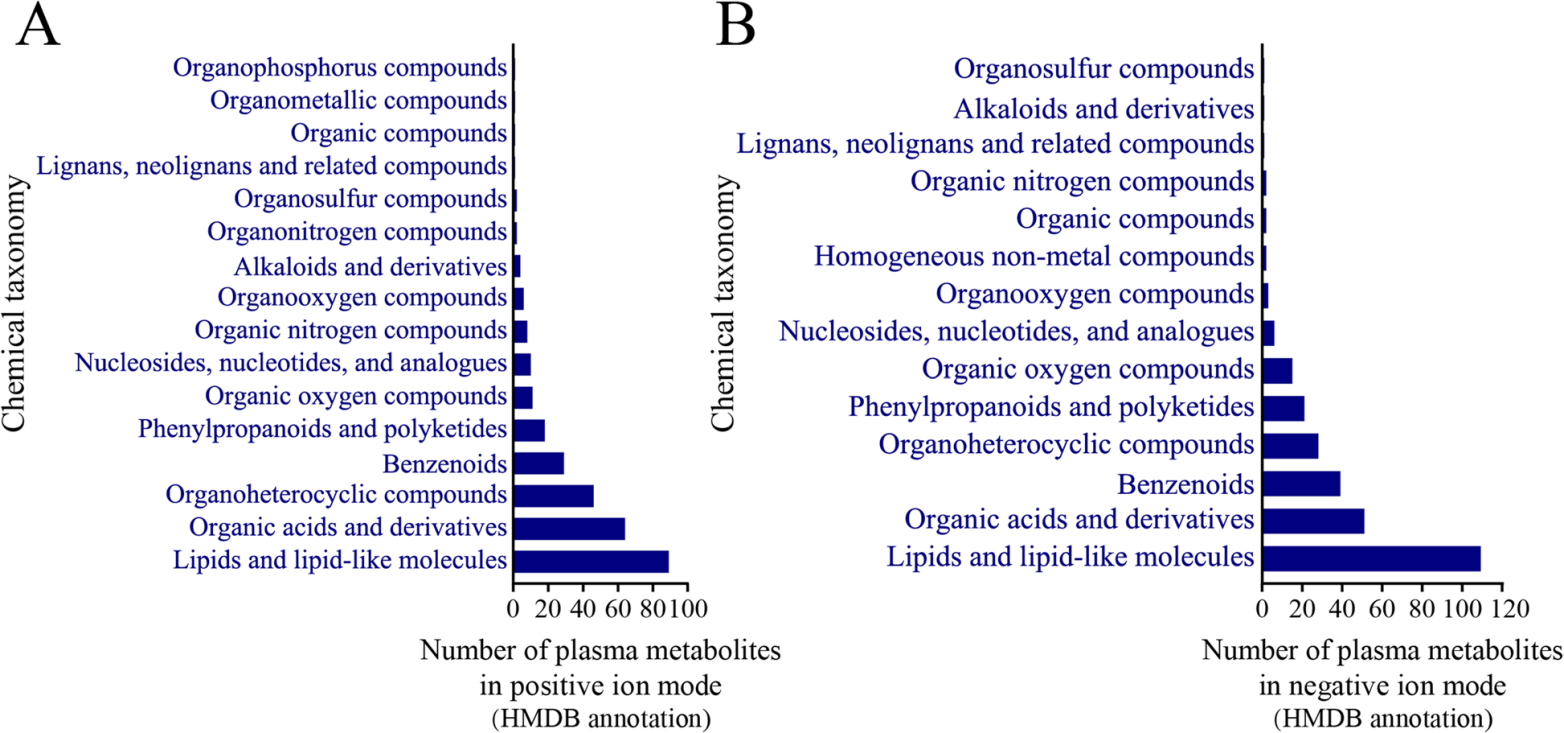
Classifications of plasma metabolites in the positive (**A**) and negative (**B**) ion modes

**Fig. 2:**
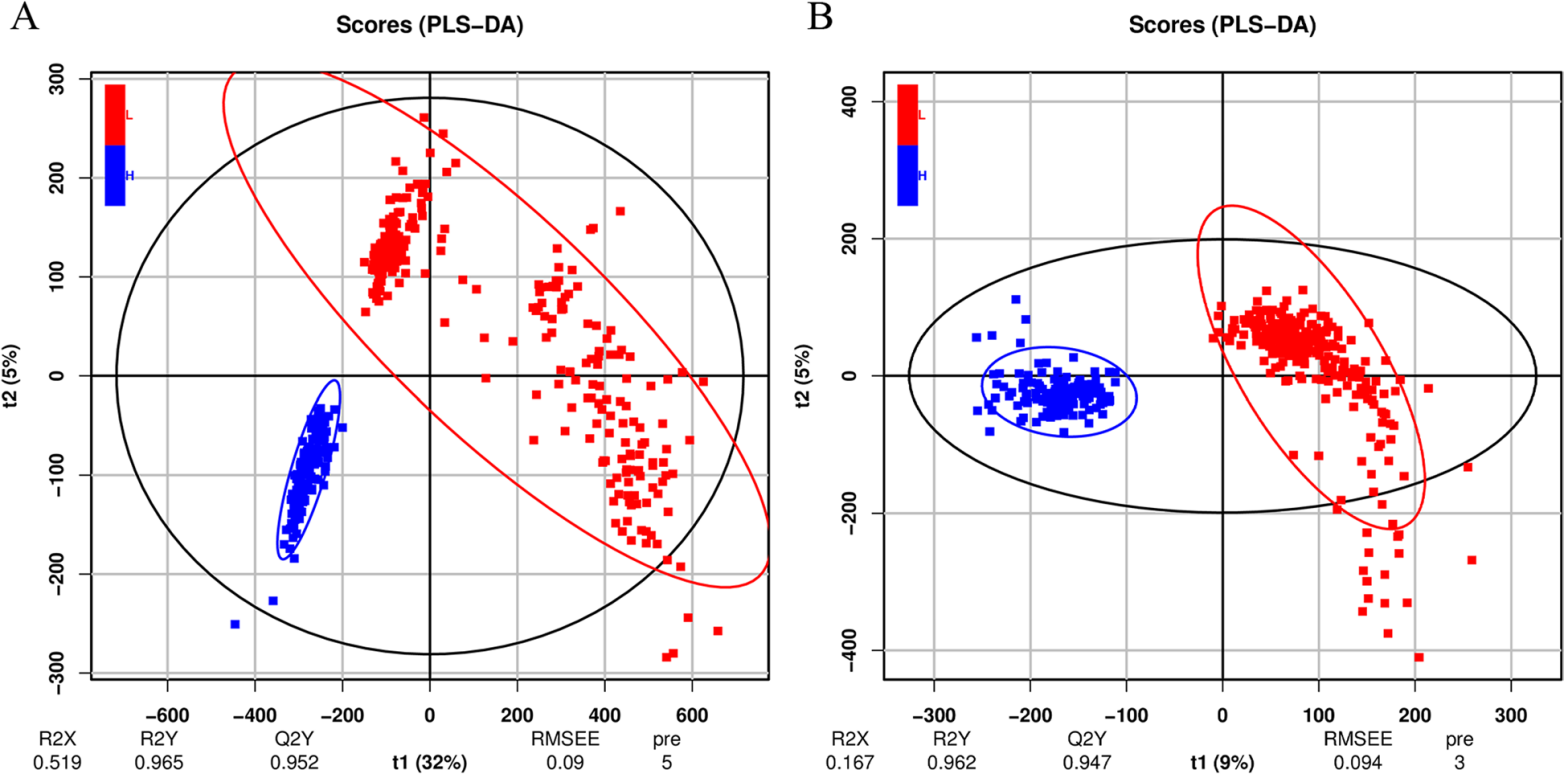
PLS-DA score plots based on plasma metabolites in the positive (**A**) and negative (**B**) ion modes. Ellipse represents the 95% confidence interval of the lean lines (red) and fat lines (blue)

**Fig. 3:**
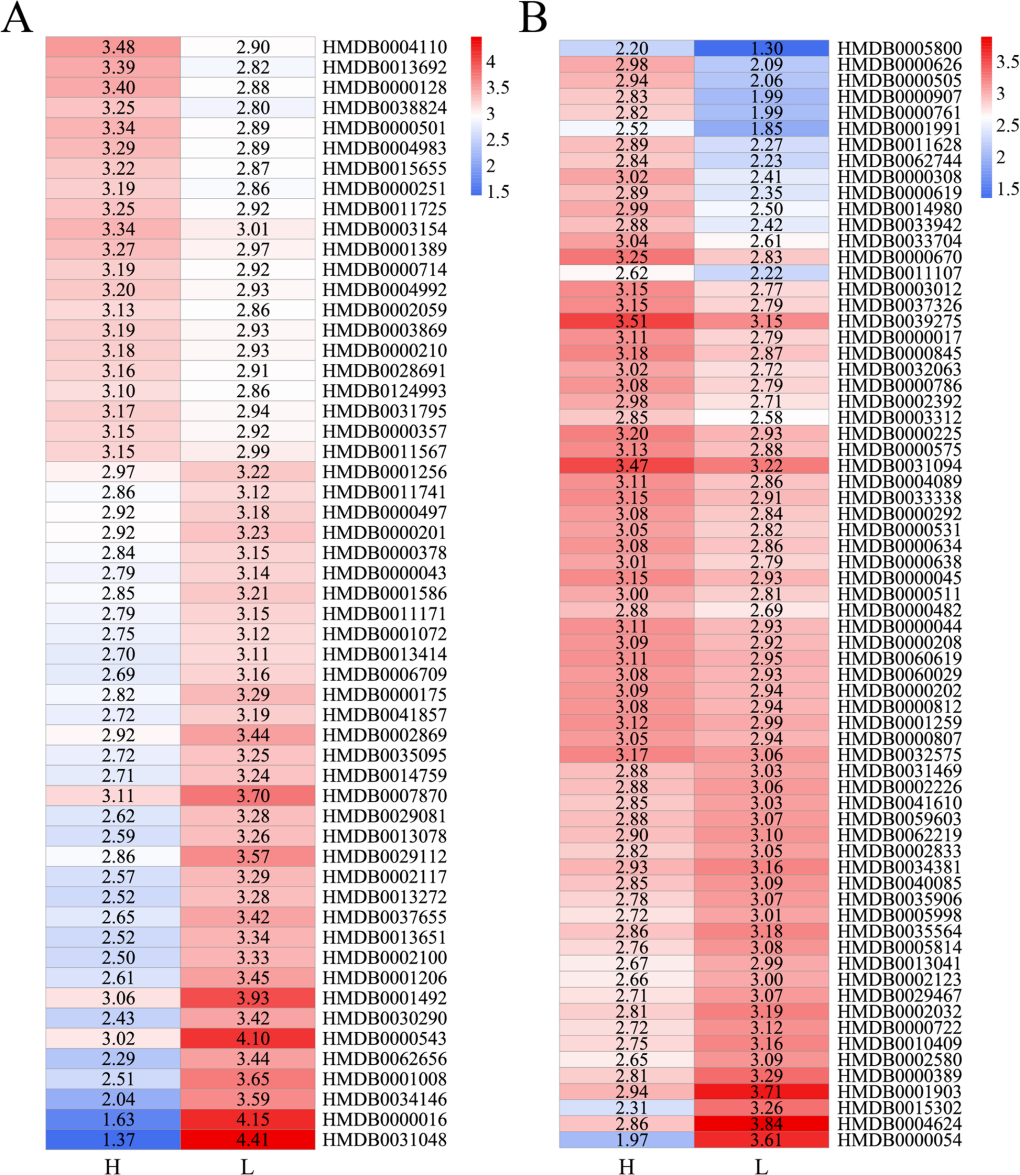
Heatmap of differentially expressed plasma metabolites in the positive (**A**) and negative (**B**) ion modes. The numbers in the grid are expressed as the log10 of the plasma concentrations of metabolites. HMDB_ID is the serial number for the metabolites established by the HMDB

In this study, a total of 941 and 882 *m/z* features in the positive and negative ion modes were found to match the search databases, respectively (Table 1). These features were annotated using the HMDB, and 284 and 272 metabolites were finally identified in the positive and negative ion modes, respectively, to be used for subsequent analysis. The metabolites, such as lipids and lipid-like molecules, lignans, neolignans and related compounds, nucleosides, nucleotides and analogs, alkaloids and derivatives, organic compounds, phenylpropanoids, and polyketides, were identified, while the lipids and lipid-like molecules comprised the most abundant of all the metabolites (Fig. 1). The results of PLS-DA of all the plasma metabolites indicated that the fat and lean broilers were divided into two independent groups in both the positive and negative ion modes, indicating differences in the metabolic levels between the fat and lean chickens (Fig. 2). The parameters of PLS-DA model in the positive ion mode, including R^2^X, R^2^Y, and Q^2^Y, were established as 0.519, 0.965, and 0.952, respectively. The R^2^X, R^2^Y, and Q^2^Y in the negative ion mode were established as 0.167, 0.962, and 0.947, respectively. The concentrations of the differentially expressed plasma metabolites between the lean and fat birds were shown in Fig. 3. In the positive ion mode, the concentrations of 55 plasma metabolites were found to be significantly different between the lean and fat birds. Of these, 21 metabolites were found to have significantly higher concentrations in the fat birds than in the lean birds, whereas 34 metabolites were found to have significantly lower concentrations in the fat birds than in the lean birds (*P* < 0.05). In the negative ion mode, the concentrations of 69 plasma metabolites were found to be significantly different between the lean and fat birds. Of these, the concentrations of 45 metabolites were significantly higher in the fat birds than in the lean birds, whereas 24 metabolites were found to have significantly lower concentrations in the fat birds than in the lean birds (*P* < 0.05).

**Table 1:** Summary of features identified by analyzing the non-targeted plasma metabolomics

Ion mode	The mass spectral database				Summary
	MzCloud	MzVault	MassList	Chemspider	
Positive	158	112	142	878	941
Negative	133	85	153	842	882

### Estimations of the genetic parameters of the differentially expressed plasma metabolites

A large number of differentially expressed plasma metabolites have been identified between the lean and fat lines. However, whether the expression levels of these metabolites can be related to FE traits is unknown, while heritability of differentially expressed plasma metabolites is also unknown. Thus, the genetic and phenotypic correlation coefficients were estimated between differentially expressed plasma metabolites and FE trait indices. Among these differentially expressed metabolites, the genetic and phenotypic correlation coefficients between 21 positive metabolites or 27 negative metabolites and FE trait indices could not be calculated due to no converge. The genetic parameters of the remaining 34 metabolites in the positive ion mode were shown in Table 2, while the remaining 42 metabolites in the negative ion mode were shown in Table 3.

**Table 2:** Genetic parameters of the differentially expressed plasma metabolites in the positive ion mode^a^

Metabolite	Common name	Heritability	Genetic correlations		Phenotypic correlations	
			FCR	RFI	FCR	RFI
HMDB0001008	Biliverdin	0.70 ± 0.13	0 ± 0.21	–0.01 ± 0.18	–0.03 ± 0.06	–0.03 ± 0.06
HMDB0029112	Tyrosyl-phenylalanine	0.57 ± 0.13	0 ± 0.22	–0.11 ± 0.19	–0.02 ± 0.05	–0.04 ± 0.06
HMDB0003154	Canthaxanthin	0.38 ± 0.12	–0.48 ± 0.22	–0.45 ± 0.20	–0.09 ± 0.05	–0.13 ± 0.05
HMDB0000501	7-Ketocholesterol	0.36 ± 0.13	0.41 ± 0.25	0.54 ± 0.22	0.07 ± 0.05	0.11 ± 0.05
HMDB0004983	Dimethyl sulfone	0.33 ± 0.12	0.40 ± 0.26	0.30 ± 0.23	–0.04 ± 0.05	0 ± 0.05
HMDB0038824	Hypoletin 8-gentiobioside	0.31 ± 0.13	0.35 ± 0.29	0.32 ± 0.26	–0.05 ± 0.05	–0.05 ± 0.05
HMDB0000210	Pantothenic acid	0.29 ± 0.11	0.16 ± 0.26	0.20 ± 0.23	0.16 ± 0.05	0.15 ± 0.05
HMDB0011567	Monoolein	0.29 ± 0.12	–0.38 ± 0.26	–0.27 ± 0.24	–0.11 ± 0.05	–0.05 ± 0.05
HMDB0000251	Taurine	0.28 ± 0.12	–0.15 ± 0.28	0.04 ± 0.25	0.04 ± 0.05	0.03 ± 0.05
HMDB0004992	Benzocaine	0.27 ± 0.11	0.12 ± 0.28	0.03 ± 0.25	0.06 ± 0.05	0 ± 0.05
HMDB0000714	Hippuric acid	0.25 ± 0.11	0.05 ± 0.28	–0.10 ± 0.25	0.04 ± 0.05	–0.01 ± 0.05
HMDB0003869	Epsilon-(gamma-glutamyl)-lysine	0.25 ± 0.11	0.19 ± 0.27	0.42 ± 0.21	0.14 ± 0.05	0.20 ± 0.05
HMDB0000128	Guanidineacetic acid	0.24 ± 0.11	–0.20 ± 0.29	0.06 ± 0.26	–0.04 ± 0.05	0.01 ± 0.05
HMDB0000357	beta Hydroxybutyrate	0.23 ± 0.10	0.25 ± 0.27	0.21 ± 0.25	0.16 ± 0.05	0.14 ± 0.05
HMDB0011741	gamma-Glutamyltyrosine	0.22 ± 0.10	–0.42 ± 0.26	–0.39 ± 0.25	–0.19 ± 0.05	–0.18 ± 0.05
HMDB0029081	Tryptophyl-glutamine	0.19 ± 0.10	0.28 ± 0.30	0.30 ± 0.27	0.04 ± 0.05	0.03 ± 0.05
HMDB0006709	Coenzyme Q2	0.16 ± 0.10	0.11 ± 0.33	0.17 ± 0.30	–0.02 ± 0.05	–0.03 ± 0.05
HMDB0000175	IMP	0.16 ± 0.10	–0.24 ± 0.33	–0.14 ± 0.30	0 ± 0.05	–0.01 ± 0.05
HMDB0028691	*L*-Alanyl-*L*-leucine	0.15 ± 0.11	–0.56 ± 0.37	–0.03 ± 0.31	0.06 ± 0.05	0.06 ± 0.05
HMDB0013651	2-(14,15-Epoxyeicosatrienoyl) glycerol	0.15 ± 0.09	0.25 ± 0.33	0.26 ± 0.31	–0.03 ± 0.05	–0.02 ± 0.05
HMDB0000378	2-Methylbutyroylcarnitine	0.14 ± 0.11	0.72 ± 0.33	0.74 ± 0.35	0.20 ± 0.05	0.16 ± 0.05
HMDB0015655	Virilon	0.13 ± 0.11	0.43 ± 0.45	0.37 ± 0.41	–0.08 ± 0.05	–0.09 ± 0.05
HMDB0011725	Sulfosalicylic acid	0.13 ± 0.09	0.38 ± 0.36	0.22 ± 0.32	0.05 ± 0.05	0.04 ± 0.05
HMDB0011171	gamma-Glu-Leu	0.11 ± 0.09	0.08 ± 0.38	0.17 ± 0.35	0.01 ± 0.05	0.01 ± 0.05
HMDB0013272	N-Lauroylglycine	0.11 ± 0.09	–0.02 ± 0.39	0.08 ± 0.36	–0.01 ± 0.05	–0.02 ± 0.05
HMDB0000016	Desoxycortone	0.11 ± 0.09	0.29 ± 0.40	0.35 ± 0.36	–0.02 ± 0.05	–0.02 ± 0.05
HMDB0001072	Ubiquinone-10	0.08 ± 0.09	–0.47 ± 0.45	-0.59 ± 0.42	0.05 ± 0.05	0.04 ± 0.05
HMDB0062656	Linoleamide	0.08 ± 0.09	0.37 ± 0.46	0.44 ± 0.42	–0.04 ± 0.05	–0.04 ± 0.05
HMDB0000201	Acetyl-*L*-carnitine	0.08 ± 0.09	–0.01 ± 0.42	0.13 ± 0.38	0.18 ± 0.05	0.13 ± 0.05
HMDB0031048	Avocadyne 1-acetate	0.08 ± 0.09	0.28 ± 0.46	0.40 ± 0.43	–0.03 ± 0.05	–0.03 ± 0.05
HMDB0002100	Palmitoyl ethanolamide	0.08 ± 0.09	0.26 ± 0.47	0.40 ± 0.45	–0.04 ± 0.05	–0.04 ± 0.05
HMDB0001586	Glucose 1-phosphate	0.07 ± 0.08	0.22 ± 0.46	–0.10 ± 0.41	–0.02 ± 0.05	–0.03 ± 0.05
HMDB0002117	Oleamide	0.07 ± 0.08	0.28 ± 0.48	0.34 ± 0.46	–0.02 ± 0.05	–0.01 ± 0.05
HMDB0041857	Citrinin	0.06 ± 0.08	0.51 ± 0.56	0.67 ± 0.53	–0.04 ± 0.05	–0.05 ± 0.05

^a^Values are expressed as estimates ± SE. Metabolites with zero or near-zero heritability estimates were not listed

**Table 3:** Genetic parameters of the differentially expressed plasma metabolites in the negative ion mode^a^

Metabolite	Common name	Heritability	Genetic correlations		Phenotypic correlations	
			FCR	RFI	FCR	RFI
HMDB0031469	Asarone	0.59 ± 0.13	–0.12 ± 0.22	–0.01 ± 0.19	–0.11 ± 0.05	–0.11 ± 0.06
HMDB0000225	2-Oxoadipic acid	0.58 ± 0.13	0.44 ± 0.20	0.46 ± 0.17	0.12 ± 0.05	0.19 ± 0.06
HMDB0004089	2-(Formylamino)benzoic acid	0.54 ± 0.13	–0.14 ± 0.23	–0.25 ± 0.20	–0.11 ± 0.05	–0.16 ± 0.06
HMDB0000670	*L*-Homoarginine	0.53 ± 0.13	0.34 ± 0.23	0.37 ± 0.20	–0.03 ± 0.06	0.09 ± 0.06
HMDB0035906	(+)-Alantolactone	0.51 ± 0.12	0.07 ± 0.23	0.06 ± 0.20	–0.04 ± 0.05	–0.05 ± 0.06
HMDB0033338	Mollicellin F	0.43 ± 0.13	0.38 ± 0.23	0.25 ± 0.21	0.18 ± 0.05	0.19 ± 0.05
HMDB0002833	Testosterone	0.43 ± 0.12	–0.58 ± 0.20	–0.58 ± 0.18	–0.22 ± 0.05	–0.23 ± 0.05
HMDB0000045	Adenosine 5'-monophosphate	0.42 ± 0.12	0.51 ± 0.23	0.30 ± 0.20	0.18 ± 0.05	0.18 ± 0.05
HMDB0029467	Eugenitin	0.42 ± 0.13	–0.02 ± 0.26	–0.09 ± 0.22	0.03 ± 0.05	0 ± 0.06
HMDB0000017	4-Pyridoxic acid	0.41 ± 0.13	0.28 ± 0.26	0.12 ± 0.23	0.01 ± 0.05	–0.04 ± 0.06
HMDB0000531	3-Hydroxyvaleric acid	0.41 ± 0.13	–0.23 ± 0.26	0.18 ± 0.23	0.07 ± 0.05	0.11 ± 0.06
HMDB0005814	Testate	0.38 ± 0.12	–0.22 ± 0.26	–0.09 ± 0.23	0.02 ± 0.05	0.01 ± 0.06
HMDB0002226	Adrenic acid	0.37 ± 0.12	–0.68 ± 0.23	–0.38 ± 0.21	–0.15 ± 0.05	–0.17 ± 0.05
HMDB0000575	*DL*-Homocystine	0.36 ± 0.13	0.10 ± 0.27	0.10 ± 0.24	0.16 ± 0.05	0.15 ± 0.05
HMDB0062219	(13Z,16Z)-Docosadienoic acid	0.33 ± 0.12	–0.67 ± 0.26	–0.42 ± 0.23	–0.08 ± 0.05	–0.11 ± 0.05
HMDB0060029	5-(3',4',5'-Trihydroxyphenyl)-gamma-valerolactone-O-methyl-O-sulphate	0.32 ± 0.13	0.19 ± 0.28	0.13 ± 0.25	0.17 ± 0.05	0.09 ± 0.05
HMDB0003012	Aniline	0.28 ± 0.11	–0.14 ± 0.28	–0.31 ± 0.24	–0.02 ± 0.05	–0.08 ± 0.05
HMDB0000634	Mesaconic acid	0.28 ± 0.11	–0.09 ± 0.29	0.22 ± 0.26	–0.03 ± 0.05	0.02 ± 0.05
HMDB0000054	Bilirubin	0.28 ± 0.10	–0.04 ± 0.28	0.03 ± 0.24	0 ± 0.05	–0.01 ± 0.05
HMDB0000308	3b-Hydroxy-5-cholenoic acid	0.24 ± 0.12	0.39 ± 0.31	0.66 ± 0.28	0.03 ± 0.05	0.06 ± 0.05
HMDB0002032	8-Oxoguanine	0.24 ± 0.12	–0.11 ± 0.32	0.01 ± 0.27	0 ± 0.05	0.03 ± 0.05
HMDB0000292	Xanthine	0.21 ± 0.11	0.03 ± 0.33	–0.12 ± 0.28	–0.02 ± 0.05	–0.06 ± 0.05
HMDB0001903	Calcitriol	0.21 ± 0.11	–0.38 ± 0.32	–0.02 ± 0.29	–0.05 ± 0.05	–0.02 ± 0.05
HMDB0039275	Gingerdione	0.19 ± 0.10	–0.31 ± 0.32	–0.31 ± 0.28	–0.01 ± 0.05	0.02 ± 0.05
HMDB0000807	3-Phosphoglyceric acid	0.19 ± 0.11	–0.39 ± 0.35	–0.21 ± 0.30	–0.02 ± 0.05	0.03 ± 0.05
HMDB0000044	*L*-Ascorbate	0.17 ± 0.10	–0.58 ± 0.31	–0.37 ± 0.28	–0.08 ± 0.05	–0.08 ± 0.05
HMDB0000208	2-Oxoglutaric acid	0.17 ± 0.11	–0.07 ± 0.35	0.07 ± 0.31	0.02 ± 0.05	–0.01 ± 0.05
HMDB0000812	N-Acetylaspartic acid	0.15 ± 0.11	–0.78 ± 0.37	–0.55 ± 0.34	–0.04 ± 0.05	–0.06 ± 0.05
HMDB0001991	7-Methylxanthine	0.14 ± 0.11	–0.19 ± 0.40	0.51 ± 0.33	–0.11 ± 0.05	–0.06 ± 0.05
HMDB0000786	Oxypurinol	0.12 ± 0.11	–0.06 ± 0.42	–0.28 ± 0.35	–0.10 ± 0.05	–0.14 ± 0.05
HMDB0005998	20-Hydroxy-(5Z,8Z,11Z,14Z)-eicosatetraenoic acid	0.12 ± 0.09	–0.67 ± 0.37	–0.58 ± 0.33	–0.10 ± 0.05	–0.13 ± 0.05
HMDB0033704	Hexose	0.11 ± 0.10	0.12 ± 0.43	–0.04 ± 0.38	0 ± 0.05	0.02 ± 0.05
HMDB0041610	Phenylacetaldehyde	0.10 ± 0.10	–0.38 ± 0.42	–0.23 ± 0.38	–0.16 ± 0.05	–0.12 ± 0.05
HMDB0002580	Taurolithocholic acid sulfate	0.10 ± 0.09	0.07 ± 0.44	0.26 ± 0.38	0.04 ± 0.05	0.03 ± 0.05
HMDB0015302	Aurorix	0.09 ± 0.09	–0.06 ± 0.44	0.31 ± 0.39	–0.02 ± 0.05	0.01 ± 0.05
HMDB0000202	Methylmalonic acid	0.08 ± 0.09	0.13 ± 0.50	–0.45 ± 0.40	0 ± 0.05	–0.05 ± 0.05
HMDB0000389	2'-Deoxysepiapterin	0.07 ± 0.09	0.63 ± 0.57	0.21 ± 0.45	–0.02 ± 0.05	–0.01 ± 0.05
HMDB0000511	Decanoic acid	0.06 ± 0.09	0.31 ± 0.54	0.73 ± 0.48	–0.01 ± 0.05	0.03 ± 0.05
HMDB0035564	6,8-Tricosanedione	0.06 ± 0.08	-	–0.49 ± 0.59	-	–0.02 ± 0.05
HMDB0011628	Enoxolone	0.05 ± 0.10	0.67 ± 1.02	-	-	-
HMDB0040085	2,5-Dipropyl-4-methylthiazole	0.05 ± 0.10	0.38 ± 0.52	0.03 ± 0.53	0.12 ± 0.05	0.02 ± 0.05
HMDB0004624	3b,7b-Dihydroxy-5-androsten-17-one	0.05 ± 0.08	–0.24 ± 0.57	–0.40 ± 0.49	–0.03 ± 0.05	0 ± 0.05

^a^Values are expressed as estimates ± SE. The “-” in the table refers to the values that were not converging in solving the animal model and were not calculated. Metabolites with zero or near-zero heritability estimates were not listed

In the positive ion mode (Table 2), 14 differentially expressed plasma metabolites were found to have higher positive genetic correlations with the FE trait indices (*r*_g_ ≥ 0.3), whereas 5 differentially expressed plasma metabolites were found to have higher negative genetic correlations with the FE trait indices (*r*_g_ ≤ –0.3). The differentially expressed plasma metabolites and the FE trait indices showed relatively lower phenotypic correlation coefficients (–0.19 ≤ *r*_p_ ≤ 0.20). Among the differentially expressed plasma metabolites, the heritability estimates of biliverdin and tyrosyl-phenylalanine were high (0.57 ≤ *h*^2^ ≤ 0.70). The heritability estimates of canthaxanthin, 7-ketocholesterol, dimethyl sulfone, hypoletin 8-gentiobioside, pantothenic acid, monoolein, taurine, benzocaine, hippuric acid, epsilon-(gamma-glutamyl)-lysine, guanidine acetic acid, beta-hydroxybutyrate, and gamma-glutamyltyrosine were found to be moderate (0.22 ≤ *h*^2^ ≤ 0.38).

In the negative ion mode (Table 3), 11 differentially expressed plasma metabolites had higher positive genetic correlations with the FE trait indices (*r*_g_ ≥ 0.3), whereas 14 differentially expressed plasma metabolites demonstrated higher negative genetic correlations with the FE trait indices (*r*_g_ ≤ –0.3). Relatively lower phenotypic correlation coefficients were observed between the differentially expressed plasma metabolites and the FE trait indices (–0.23 ≤ *r*_p_ ≤ 0.19). Among the differentially expressed plasma metabolites, the heritability estimates of asarone, 2-oxoadipic acid, 2-(formal amino) benzoic acid, *L*-homoarginine, (+)-alantolactone, mollicellin F, testosterone, adenosine 5′-monophosphate, eugenitin, 4-pyridoxic acid, and 3-hydroxyvaleric acid were found to be high (0.41 ≤ *h*^2^ ≤ 0.59). The heritability estimates of testate, adrenic acid, *DL*-homocystine, (13Z,16Z)-docosadienoic acid, 5-(3′,4′,5′-trihydroxyphenyl)-gamma-valerolactone-O-methyl-O-sulphate, aniline, mesaconic acid, bilirubin, 3b-hydroxy-5-cholenoic acid, 8-oxoguanine, xanthine, and calcitriol were found to be moderate (0.21 ≤ *h*^2^ ≤ 0.38).

## Discussion

The NEAUHLF lines were successfully bred, and the AFP of broilers in the fat line was found to be 11.02 times higher than in the lean line after selecting the 23^rd^ generation [11]. A previous study showed that FE traits changed significantly with the selection of AFP. The FCR and RFI in the lean line were 2.41 ± 0.01 and –160.08 ± 7.96 g, respectively, while those in the fat line were 2.81 ± 0.01 and 296.3 ± 11.05 g, respectively [11]. Besides, the FE trait indices showed high positive genetic correlations with the abdominal fat weight (AFW) and AFP in the NEAUHLF lines (from 0.49 to 0.58) [11]. These results indicated that the NEAUHLF lines could be used as high-FE and low-FE animal models. Based on our previous study, the present study further explored the differences in the plasma metabolic levels between the NEAUHLF lines using the same experimental chickens, and identified the potentially useful metabolite biomarkers for selecting the "food-saving broilers".

Firstly, the PLS-DA method was used for identifying the differentially expressed plasma metabolites between the lean and fat birds. PLS-DA, as a supervised and multivariate analysis approach for dimensionality reduction, was widely used for metabolomics researchs and has been established as a standard high-dimensional data analysis method [18, 19]. The value of Q^2^Y could explain the predictive ability of the supervised PLS-DA model and was found to be better if it was close to 1 theoretically [20]. In the present study, the Q^2^Y was 0.952 in the positive ion mode, and 0.947 in the negative ion mode (Fig. 2), indicating that PLS-DA model was successfully established. The results of the PLS-DA showed that the lean and fat birds were separated into two independent groups (Fig. 2), identifying 124 significantly and differentially expressed metabolites between the lean and fat birds.

The genetic and phenotypic correlations between the metabolites and the FE trait indices were estimated to identify the differentially expressed metabolites associated with the FE traits. In the positive ion mode, 19 metabolites were found to have high genetic correlations with the FE traits indices (|*r*_g_| ≥ 0.30) (Table 2). In negative ion mode, 25 metabolites were found to have high genetic correlations with the FE traits indices (|*r*_g_| ≥ 0.30) (Table 3). In addition, among the differentially expressed plasma metabolites, 15 metabolites in the positive ion mode (Table 2) and 23 metabolites in the negative ion mode (Table 3) were found to display moderate to high heritability estimates. To date, reports on estimating the genetic parameters of plasma metabolites in birds are few.

**Fig. 4:**
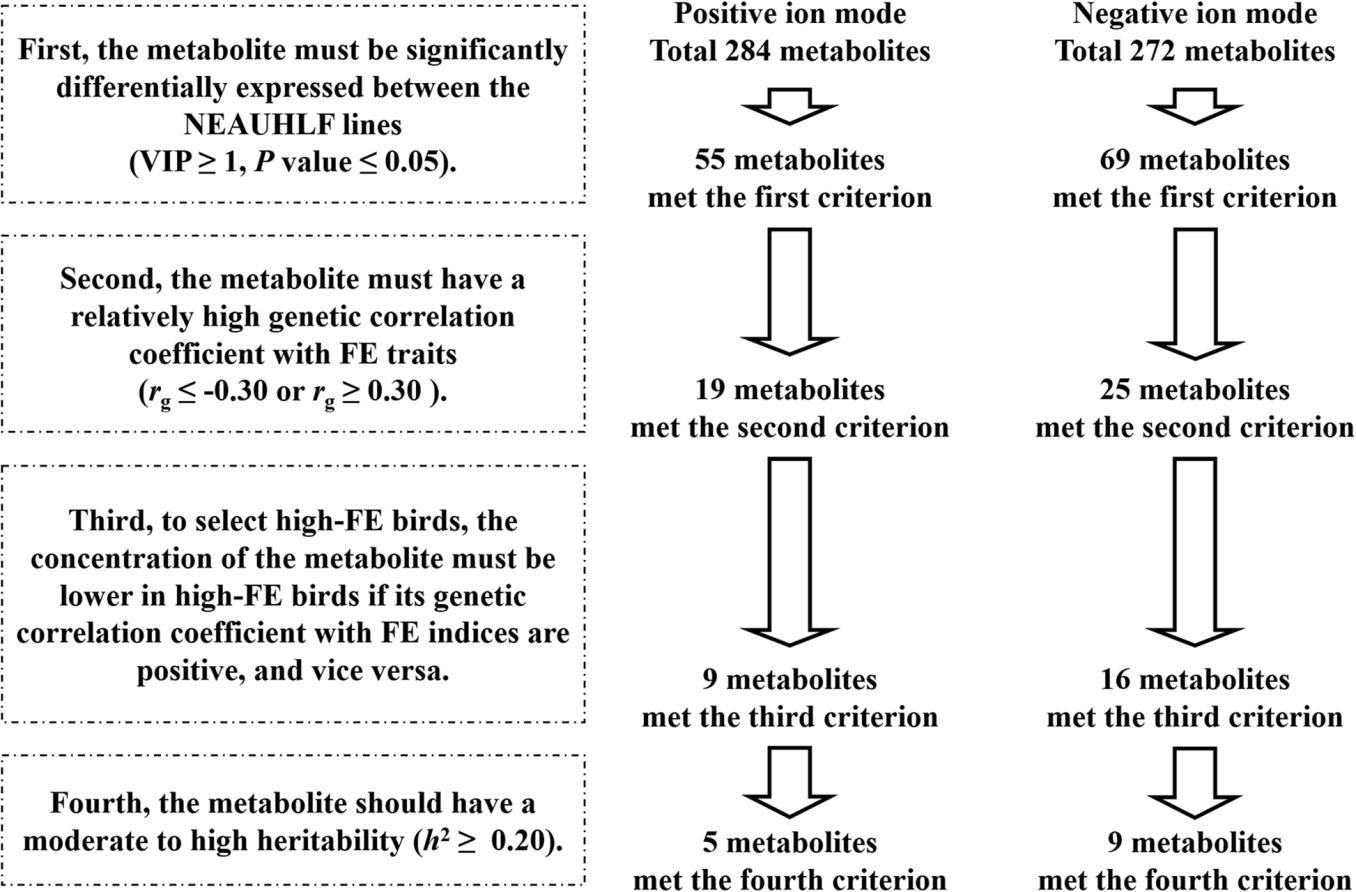
Pipeline for the selection of the metabolite biomarkers

The objective of this study was to identify the metabolites that could be used as biomarkers for selecting broilers with high FE. Accordingly, a pipeline containing four criteria was established for determining whether a given metabolite could be used as a marker (Fig. 4). First, the metabolite concentration between the lean and fat birds must be significantly different. Second, the genetic correlation coefficient between the FE trait indices and the metabolite concentration should be relatively high (genetic correlation threshold: |*r*_g_| ≥ 0.30). Third, the concentration of the metabolite must be lower in the high-FE birds (lean line), if the genetic correlation coefficients with the FE trait indices were positive, and vice versa. Fourth, the heritability of the metabolite concentration should be moderate to high (heritability threshold: *h*^2^ ≥ 0.20). In the positive and negative ion modes between the lean and fat birds, 124 metabolites were found to have significantly different concentrations, meeting the first criterion. Among these 124 metabolites, 44 were found to have relatively high genetic correlations with the FE trait indices, meeting the second criterion. Of these 44 metabolites, 25 met the third criterion. Finally, of these 25 metabolites, 14 met the fourth criterion, which was shown in Table 4 and Fig. 4. In the positive ion mode, five metabolites including 7-ketocholesterol, dimethyl sulfone, hypoletin 8-gentiobioside, epsilon-(gamma-glutamyl)-lysine, and gamma-glutamyltyrosine were found to meet all the four criteria. In the negative ion mode, nine metabolites including 2-oxoadipic acid, *L*-homoarginine, mollicellin F, testosterone, adenosine 5′-monophosphate, adrenic acid, (13Z,16Z)-docosadienoic acid, 3b-hydroxy-5-cholenoic acid, and calcitriol were found to meet all four criteria. Therefore, these 14 metabolites were presumed to have the potential to be used as the biomarkers for selecting "food-saving broilers".

**Table 4:** Summary of 14 metabolite biomarkers for selecting the high-FE birds

Ion mode	Common name	Expression		Genetic correlations		Heritability
		Fat line	Lean line	FCR	RFI	
Positive	7-Ketocholesterol	2180.27	782.69	0.41 ± 0.25	0.54 ± 0.22	0.36 ± 0.13
	Dimethyl sulfone	1935.83	770.09	0.40 ± 0.26	0.30 ± 0.23	0.33 ± 0.12
	Hypoletin 8-gentiobioside	1783.14	625.76	0.35 ± 0.29	0.32 ± 0.26	0.31 ± 0.13
	Epsilon-(gamma-glutamyl)-lysine	1531.94	851.93	0.19 ± 0.27	0.42 ± 0.21	0.25 ± 0.11
	gamma-Glutamyltyrosine	720.39	1309.21	–0.42 ± 0.26	–0.39 ± 0.25	0.22 ± 0.10
Negative	2-Oxoadipic acid	1580.83	855.62	0.44 ± 0.20	0.46 ± 0.17	0.58 ± 0.13
	*L*-Homoarginine	1762.28	670.93	0.34 ± 0.23	0.37 ± 0.20	0.53 ± 0.13
	Mollicellin F	1418.72	806.64	0.38 ± 0.23	0.25 ± 0.21	0.43 ± 0.13
	Testosterone	660.15	1114.50	–0.58 ± 0.20	–0.58 ± 0.18	0.43 ± 0.12
	Adenosine 5'-monophosphate	1399.03	856.74	0.51 ± 0.23	0.30 ± 0.20	0.42 ± 0.12
	Adrenic acid	766.70	1155.87	–0.68 ± 0.23	–0.38 ± 0.21	0.37 ± 0.12
	(13Z,16Z)-Docosadienoic acid	790.03	1248.22	–0.67 ± 0.26	–0.42 ± 0.23	0.33 ± 0.12
	3b-Hydroxy-5-cholenoic acid	1045.31	257.59	0.39 ± 0.31	0.66 ± 0.28	0.24 ± 0.12
	Calcitriol	869.84	5096.83	–0.38 ± 0.32	–0.02 ± 0.29	0.21 ± 0.11

The published literature was searched, and the biological functions of 14 metabolites were summarized to rationally explain the physiological basis of the FE traits. However, among the 14 metabolites, no reports were available on the physiological functions of 4 metabolites, including hypoletin 8-gentiobioside, mollicellin F, (13Z,16Z)-docosadienoic acid, and 3b-hydroxy-5-cholenoic acid. Therefore, the effects of these 4 metabolites on FE traits of broilers could not be speculated. The remaining 10 metabolites were suggested to be biomarkers after analyzing the relationship between these metabolites and FE traits.

7-Ketocholesterol is a pro-inflammatory oxysterol that possibly activates several kinase signaling pathways via multiple transcription factors for inducing the cytokines and intracellular effectors causing cell death [21]. 7-Ketocholesterol damages the vascular endothelial cells by inducing inflammatory responses and apoptosis, elevating the risk of cardiovascular diseases [22]. Recent studies have shown the inflammatory responses to reduce FE in animals. In pigs, the inflammatory reaction could reduce FE [23]; a similar phenomenon was observed in cattle [24]. The fat line in this study had a higher concentration of 7-ketocholesterol than that in the lean line. A previous study showed that the FE in the fat line was significantly lower than that in the lean line [11]. Based on the reports from other studies and the results of this study, the fat line was suggested to have a stronger inflammatory response than the lean line and, therefore, a lower FE.

Dimethyl sulfone, also known as methylsulfonylmethane, is an organic sulfur-containing compound naturally occurring in various grains and animal tissues [25]. Dimethyl sulfone is an oxidative stress marker [26], and oxidative stress reportedly causes inflammatory responses [27, 28]. In beef cattle, positive correlations existed between the plasma dimethyl sulfone and the FE trait indices [29], which was consistent with the results of the present study (Table 4). In the present study, the fat line was found to have a higher concentration of dimethyl sulfone than the lean line. The reports of other studies and the findings of this study suggested that the fat line had stronger oxidative stress and inflammatory response compared with the lean line and, therefore, a lower FE. As a result, selecting birds with low plasma dimethyl sulfone was suggested for improving the FE.

Epsilon-(gamma-glutamyl)-lysine, belonging to the class of organic compounds known as glutamine and derivatives, was a potential biomarker in the inflammatory reaction [30, 31]. Yang et al. and Xu et al. proposed that high-FE animals have lower inflammatory responses [32, 33]. This study reported that the fat line had a higher concentration of epsilon-(gamma-glutamyl)-lysine than the lean line, demonstrating that the fat line might have a stronger inflammatory reaction than the lean line and, therefore, a lower FE. Therefore, plasma epsilon-(gamma-glutamyl)-lysine was suggested to serve as a potential biomarker for selecting FE traits.

Gamma-glutamyltyrosine is the precursor of tyrosine (Tyr) [34]. Previous studies showed that injecting gamma-glutamyltyrosine tended to increase the plasma Tyr levels in rats [35, 36]. This study reported that the gamma-glutamyltyrosine concentration in the fat line was significantly lower than that in the lean line, suggesting that the fat birds possibly had a weaker capacity for supplying Tyr than the lean birds. Tyr possessed the anti-stress biological function [37]. Therefore, the lean birds might have strong anti-stress ability and, hence, high FE. In addition, Tyr is essential for synthesizing proteins [36], suggesting that the gamma-glutamyltyrosine content can reflect the direction of the protein metabolism in animals. Our previous study found that the breast meat percentage of the fat line was significantly lower than that of the lean line (data not shown). These findings suggested the fat birds possibly had a weaker capacity for supplying Tyr and protein synthesis ability than the lean birds and, therefore, a lower FE.

2-Oxoadipic acid is a key metabolite of tryptophan and lysine [38, 39], identified as a biomarker for acute myocardial infarction and relevant pathological changes in inflammation [40]. In this study, the fat line was found to have a higher concentration of 2-oxoadipic acid than the lean line, suggesting that the fat line might have a stronger inflammatory reaction than the lean line. Therefore, the plasma 2-oxoadipic acid was suggested to serve as the potential biomarker for selecting FE traits.

*L*-homoarginine is a nonessential cationic amino acid synthesized from arginine and lysine in vivo of bird [41]. The genome-wide association studies identified an association between the *L*-homoarginine plasma concentration and SNPs related to the *AGAT* gene [42]. Further metabolic analysis revealed that humans and mice with AGAT deficiency were inefficient in synthesizing *L*-homoarginine from *L*-arginine and *L*-lysine [42, 43]. The AGAT-deficient mice were found to exhibit decreased fat deposition, attenuated gluconeogenesis, reduced cholesterol levels, and enhanced glucose tolerance [44]. Based on these advances about *L*-homoarginine, fat deposition was found to show a positive correlation with the concentration of *L*-homoarginine. A study by Ramayo-Caldas et al. reported that lipid metabolism was associated with FE in pigs [23]. In this study, the fat line was found to have a higher concentration of *L*-homoarginine than the lean line, which was consistent with previous findings. Therefore, plasma *L*-homoarginine was suggested to serve as the potential biomarker for selecting FE traits.

Testosterone is an anabolic steroid from the androstane class of steroids [45]. It is the most important androgen, stimulating muscle growth and inhibiting lipid synthesis [46, 47]. In humans, testosterone replacement therapy reduces the body fat mass, particularly in truncal adiposity, cholesterol, and triglycerides [48]. In pigs, testosterone reduces the serum cholesterol levels [49], while in broilers, testosterone promotes the proliferation of the embryonic myoblasts and development of the skeletal muscle and inhibits the deposition of the abdominal fat [50]. Zhou et al. found that high muscle yield improved FE [51]. Ramayo-Caldas et al. found that more abdominal fat deposition reduced FE [23]. The concentration of plasma testosterone was found to be significantly lower in the fat line than in the lean line in the present study. A previous study from our lab found that the breast meat percentage was significantly lower in the fat line than in the lean line (data not shown), and the fat line was found to have a higher AFP and lower FE than the lean line [11]. Based on the reports from other studies and the results of the present study, plasma testosterone was suggested to improve FE.

Adenosine 5′-triphosphate decomposition produces adenosine 5′-diphosphate, and adenosine 5′-diphosphate decomposition produces adenosine 5′-monophosphate [52]. Under normal physiological conditions, the extracellular concentrations of adenosine 5′-triphosphate and its products were found to be considerably lower than the intracellular concentrations [53]. However, the extracellular concentrations of adenosine 5′-triphosphate and its products were found to increase markedly under inflammation [54, 55]. The fat line in this study was found to have a higher plasma concentration of adenosine 5'-monophosphate than the lean line. Based on the reports from previous studies and the results of the present study, the fat line was suggested to have a stronger inflammatory reaction and, therefore, a lower FE.

Adrenic acid, an endogenously synthesized polyunsaturated free fatty acid, can induce oxidative stress accompanied by cell death [56]. Many researchers highlighted the negative effects of adrenic acid on organisms. The adrenic acid level increased in patients with nonalcoholic fatty liver disease (NAFLD) and model mice [56]. Higher hepatic and plasma levels of free adrenic acid were found in the mouse model with hepatic steatosis, inflammation, mild fibrosis, obesity, and hypercholesterolemia [57]. However, in the present study, the concentration of plasma adrenic acid was found to be lower in the fat line than in the lean line. This result was inconsistent with the results of previous studies. Considering that the fat birds had higher FE trait indices, adrenic acid was hypothesized to possibly increase the FE. Further research is needed to investigate the mechanism by which adrenic acid negatively correlates with the indices of the FE traits.

Calcitriol, produced by vitamin D metabolism, is an active molecule of vitamin D that exerts its biological activity and functions in a variety of tissues in the body [58, 59]. Calcitriol is central to calcium and phosphate homeostasis and essentially ensures the proper development and maintenance of bone [60]. Besides, calcitriol is involved in maintaining immune homeostasis [61]. Calcitriol is used in humans for the therapeutic applications in immune dysfunction [62]. Studies on humans identified lean individuals to have significantly higher serum calcitriol levels than obese ones [63]. In Angus cattle, the genetic variation in RFI was found to be associated with the immune competence traits, suggesting that high-FE animals possibly had stronger immune competence [64]. This study found that the serum calcitriol concentration was significantly lower in the fat birds than in the lean birds. Combining the results of this study with the reports of calcitriol in both humans and animals, high-FE birds were suggested to have better immune competence.

Among 10 markers, 6 plasma metabolite markers (7-ketocholesterol, dimethyl sulfone, epsilon-(gamma-glutamyl)-lysine, 2-oxoadipic acid, adenosine 5'-monophosphate, and calcitriol) were found to be involved in inflammatory reaction and immune response. The genome-wide association study of FE in the chickens reported that the inflammatory reaction and immune response might affect FE [32]. The transcriptome analysis of the chicken breast muscle [33], duodenum [65], jejunum [66], and liver [32] revealed that both inflammatory reactions and immune response might affect FE. Based on the biological functions of metabolites, the present study found that high-FE chickens had lower inflammation and higher immunity. The immune response and inflammatory studies showed that the energy requirements of animals increased dramatically during inflammation, leading to less energy available for protein deposition and, hence, a lower FE [67]. The present findings combined with the results from previous reports indicated that reducing inflammation and improving immunity could improve the broiler FE.

## Conclusions

In summary, this study aimed to identify potential metabolite biomarkers for breeding "food-saving broilers" using the NEAUHLF lines, as an ideal animal model of FE traits for analyzing the relationship between FE traits and metabolites using the metabolomics strategy. According to the pipeline for the selection of the metabolite biomarkers established in this study, it was suggested that 10 metabolites, including 7-ketocholesterol, dimethyl sulfone, epsilon-(gamma-glutamyl)-lysine, gamma-glutamyltyrosine, 2-oxoadipic acid, *L*-homoarginine, testosterone, adenosine 5'-monophosphate, adrenic acid, and calcitriol could be used as the potential biomarkers for breeding the "food-saving broilers".

## Data Availability

The datasets from the current study are available from the corresponding author on reasonable request.

## Abbreviations

AFP: Abdominal fat percentage
AFW: Abdominal fat weight
BW4: Body weights at 4 weeks
BW7: Body weights at 7 weeks
BWG: Gain in body weights
FCR: Feed conversion ratio
FE: Feed efficiency
FI: Feed intake
HMDB: Human metabolome database
MMBW: Metabolic mid-test body weight
NAFLD: Nonalcoholic fatty liver disease
NEAUHLF: Northeast Agricultural University broiler lines divergently selected for abdominal fat content
NRC: National research council
PLS-DA: Partial least square-discriminant analysis
RFI: Residual feed intake
VIP: Variable importance in projection
VLDL: Very low-density lipoprotein
